# Transparent Pullulan/Mica Nanocomposite Coatings with Outstanding Oxygen Barrier Properties

**DOI:** 10.3390/nano7090281

**Published:** 2017-09-19

**Authors:** Ilke Uysal Unalan, Derya Boyacı, Silvia Trabattoni, Silvia Tavazzi, Stefano Farris

**Affiliations:** 1 DeFENS, Department of Food, Environmental and Nutritional Sciences—Packaging Division, University of Milan, via Celoria, 2, 20133 Milan, Italy; boyaci.derya@gmail.com; 2 Department of Food Engineering, Faculty of Engineering, İzmir University of Economics, İzmir 35330, Turkey; 3 School of Packaging, Michigan State University, East Lansing, MI 48824, USA; 4 Department of Food Engineering, Izmir Institute of Technology, İzmir 35430, Turkey; 5 Department of Materials Science, University of Milano Bicocca, via Cozzi 55, 20125 Milan, Italy; silvia.trabattoni@mater.unimib.it (S.T.); silvia.tavazzi@mater.unimib.it (S.T.); 6 INSTM, National Consortium of Materials Science and Technology, Local Unit University of Milan, via Celoria 2, 20133 Milan, Italy

**Keywords:** coefficient of friction, haze, mica, modelling, optical properties, oxygen barrier, pullulan

## Abstract

This study presents a new bionanocomposite coating on poly(ethylene terephthalate) (PET) made of pullulan and synthetic mica. Mica nanolayers have a very high aspect ratio (α), at levels much greater than that of conventional exfoliated clay layers (e.g., montmorillonite). A very small amount of mica (0.02 wt %, which is ϕ ≈ 0.00008) in pullulan coatings dramatically improved the oxygen barrier performance of the nanocomposite films under dry conditions, however, this performance was partly lost as the environmental relative humidity (RH) increased. This outcome was explained in terms of the perturbation of the spatial ordering of mica sheets within the main pullulan phase, because of RH fluctuations. This was confirmed by modelling of the experimental oxygen transmission rate (*OTR*) data according to Cussler’s model. The presence of the synthetic nanobuilding block (NBB) led to a decrease in both static and kinetic coefficients of friction, compared with neat PET (≈12% and 23%, respectively) and PET coated with unloaded pullulan (≈26% reduction in both coefficients). In spite of the presence of the filler, all of the coating formulations did not significantly impair the overall optical properties of the final material, which exhibited haze values below 3% and transmittance above 85%. The only exception to this was represented by the formulation with the highest loading of mica (1.5 wt %, which is ϕ ≈ 0.01). These findings revealed, for the first time, the potential of the NBB mica to produce nanocomposite coatings in combination with biopolymers for the generation of new functional features, such as transparent high oxygen barrier materials.

## 1. Introduction

Incorporation of two-dimensional nanomaterials as nanobuilding blocks (NBBs) in polymeric matrices paved the way for cutting-edge composites with unprecedented functional properties. Platelet-like nanoparticles in particular, such as layered silicates, have attracted much attention over the last twenty years. Layered silicate minerals include several classes and many groups that, in turn, account for different mineral species that are potentially suitable to produce nanocomposites. However, only few of these minerals (especially montmorillonite) have been widely exploited thus far [1]. Commercially available natural and organically modified montmorillonite show some disadvantages, such as limited aspect ratios (α) < 100 and high surface charge heterogeneity. This quality leads to non-uniform interlayer reactivity that hampers control over the nanoplatelets’ stiffness [2]. The bigger advantage of using synthetic clays is represented by standardized physicochemical properties. Unlike natural clays, micas that can expand with very high aspect ratios have been obtained by synthetic pathways, which eventually promote better and higher quality dispersion in the polymer matrix [1,2]. For this reason, the use of synthetic NBBs as attractive nanomaterials is rising at both the academic and industrial level. Until now, only a few studies have dealt with the use of mica in nanocomposites, including thermoset and thermoplastic polymers to improve barrier performance [3,4] and mechanical properties [2,5,6,7]. There is, however, a gap in the literature that this work aims to fill, specifically dealing with advances regarding mica-based biopolymer nanocomposite coatings.

The use of nanofiller for this generation of bionanocomposites has enormous potential for overcoming the drawbacks that are exhibited by biopolymers, such as poor mechanical and thermal properties, sensitivity to moist environments, and inadequate barrier properties to gas and vapors. However, most examples were concerned with the incorporation of the inorganic phase directly into the bulky biopolymer. The use of fillers within coatings made of biopolymers has been proposed only very recently, to produce bionanocomposite coatings that improve the properties of a plastic substrate without jeopardizing its original attributes, to optimize cost efficiency, and to reduce environmental impact [1,8]. Bionanocomposite coatings were successfully produced by using natural montmorillonite (Na^+^-MMT) [9] and graphene oxide [10] to improve the oxygen barrier properties, even at high relative humidity values, or in combination with microfibrillated cellulose (MFC) [11] and borax [12] to produce biocoatings with enhanced mechanical and permeability properties. In this study, we decided to use the NBB mica in combination with the exopolysaccharide pullulan to develop high-performance oxygen barrier materials that concurrently exhibit outstanding optical, surface, and mechanical properties. Among the variety of biopolymers, Pullulan was selected due to its unique properties, such as high flexibility, excellent transparency, good oxygen barrier properties, and non-toxicity—all of which make it a promising candidate for a next-generation of materials that are totally or partially based on renewable resources [13]. Poly(ethylene terephthalate) (PET) has been used as a plastic substrate because of its widespread use in many different applications, ranging from food packaging (e.g., liquids containers, thermoforming applications, layers for flexible packaging solutions) to energy applications (e.g., solar cells), displays, and medical/biomedical uses.

## 2. Results and Discussion

### 2.1. Morphological Characterization of Mica

**Figure 1:**
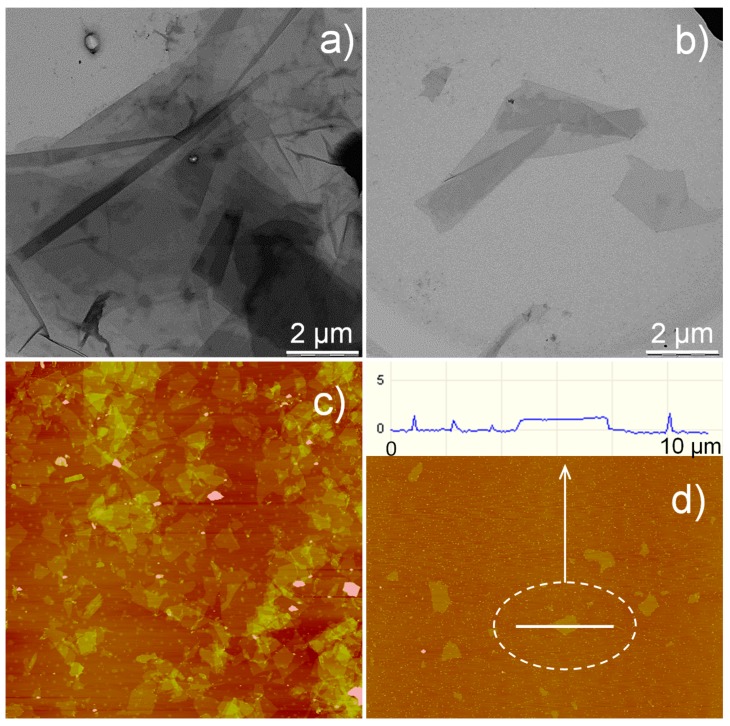
Transmission electron microscopy (TEM) images of mica nanosheets at (**a**) 0.2 wt % and (**b**) 0.02 wt %. Atomic force microscopy (AFM) height images of mica nanosheets: (**c**) at 0.2 wt % and 20 × 20 µm^2^; (**d**) at 0.02 wt % and 40 × 40 µm^2^.

Transmission electron microscopy (TEM) and atomic force microscopy (AFM) images of mica sheets are displayed in Figure 1, panels a–d. TEM allowed for the gathering of information on the degree of the exfoliation of mica sheets. As shown in Figure 1a, mica particles appeared as large overlapping sheets at the highest concentration (0.2 wt %). Dilution by one order of magnitude revealed the full exfoliation of mica to individual platelets by means of ultrasonication (Figure 1b). Quantitative information on the size of mica nanoparticles was obtained by AFM (Figure 1c,d). After collecting several images, it was possible to quantify both the width and thickness of synthetic mica, being equal to 3.6 ± 1.1 μm and 1.1 ± 0.2 nm, respectively. Eventually, an average aspect ratio (α) of 3615 ± 109 was assigned to the mica sheets, which was higher than the aspect ratio reported by [4] (α = 1064). This difference can be plausibly attributed to the different type of mica used by those authors (i.e., a synthetic mica organically modified with di-methyl di-hydrogenated tallow ammonium–chloride as intercalant).

### 2.2. Oxygen Barrier Performance

The oxygen barrier performance of bare PET, pullulan-coated PET, and pullulan/mica-coated PET films are reported in Table 1. Pullulan and pullulan nanocomposite coatings had a thickness that ranged from 0.75 μm to 0.80 μm. The deposition of the unloaded pullulan coating decreased the oxygen transmission rate (*OTR*) value of the plastic substrate (from ≈130 mL m^−2^ 24^−1^ to ≈6 mL m^−2^ 24^−1^ at 0% relative humidity (RH)). This has been explained by the tight network formed by pullulan chains due to the extensive hydrogen bonding [10,12]. The addition of mica dramatically increased the oxygen barrier performance of the pullulan coating, even at the lowest concentrations (0.02 and 0.04 wt %), thus yielding an *OTR* decrease of ≈80% in comparison to the bare pullulan coating (RH = 0%). Noticeably, from a concentration of mica of 0.06 wt % (ϕ = 0.00023) and greater, the *OTR* values of coated PET were below the instrument’s detection limit (0.01 mL m^−2^ 24^−1^). These excellent results suggest the successful exfoliation of mica nanosheets in water, mediated by ultrasonication. This process allowed the achievement of an outstanding performance, even at low loadings, due to both the “tortuosity path” and “organic/inorganic interface” effects [4].

Increasing the relative humidity of the environment in contact with the coating led to a different scenario. Starting at 30% RH, the *OTR* values of PET films coated with pullulan increased to a slight extent. The same trend was more evident at 60% and especially 90% RH, insomuch as the benefit arising from the deposition of the pullulan nanocomposite coating disappeared completely at the highest RH value. The detrimental effect of humidity on the barrier properties of pullulan nanocomposite coatings has already been described for other lamellar clays, such as natural montmorillonite [9] and, more recently, graphene oxide [10].

This effect is ascribed to the plasticizing effect of water molecules adsorbed by the polymer surface and bulk, especially in correspondence with the amorphous regions [14,15]. The tight and dense network ensuing from the cooperative adhesion forces at the biopolymer/filler interface is gradually lost, with a concurrent increase in chain mobility and free volume of the nanocomposite network. This occurs until an unconstrained diffusion of the permeant at 90% RH is observed, due to what has been previously defined as the “diluting” effect of water molecules [10]. Eventually, the physical impedance (i.e., the increase of the diffusion path) due to the high aspect ratio of mica sheets has been overcompensated by the increase in free volume and subsequent higher diffusivity of the oxygen molecules through the plasticized network.

**Figure 2:**
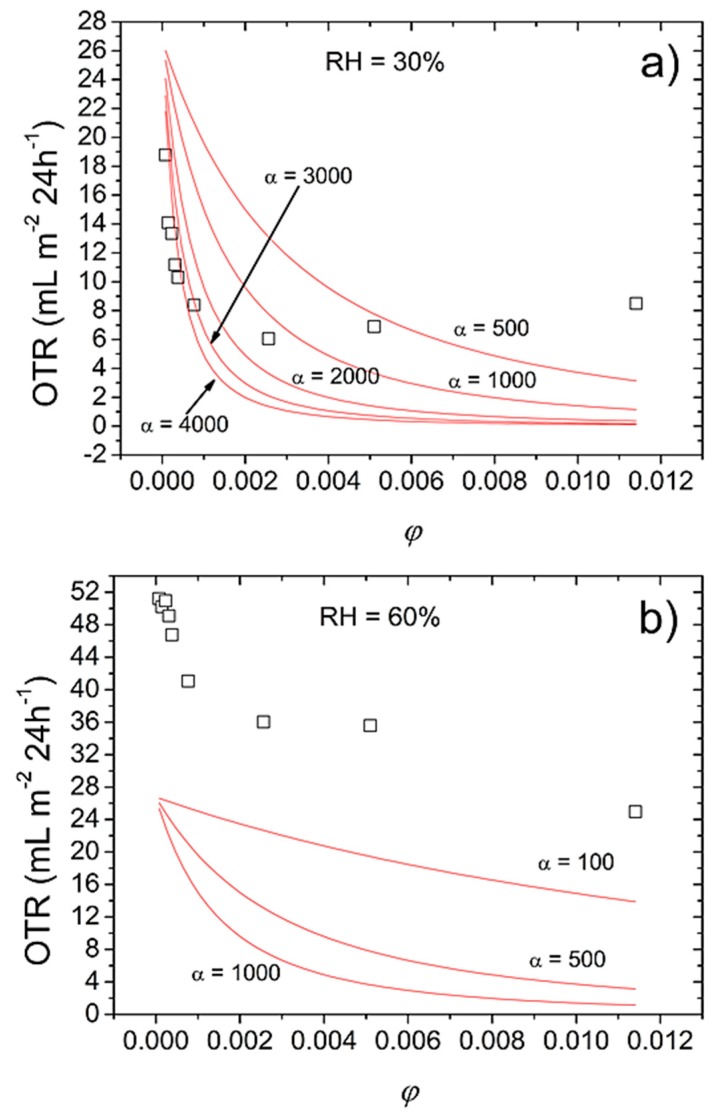
Experimental (symbols) and predicted (solid lines) *OTR* values of bionanocomposite hybrid coatings as a function of filler volume fraction (ϕ) for different aspect ratio (α) of mica platelets at (**a**) 30% RH and (**b**) 60% RH, according to Cussler’s model (Equation (1) in the text).

Permeability data has been widely modeled to acquire detailed information on the distribution and spatial organization of platy fillers in the main polymer network [16]. In turn, the outcome is a better interpretation of the ultimate O_2_-barrier performance of the final material (e.g., coated plastic film), especially when external parameters (e.g., relative humidity) rise as perturbing factors. Figure 2 depicts the experimental *OTR* data of the bionanocomposite coatings at 30% and 60% RH and 23 °C, together with a theoretical prediction based on Cussler’s permeation theoretical model. This model describes the permeation phenomenon for impermeable square platelets that are dispersed in a continuous matrix for a semi-dilute regime (i.e., αϕ >> 1) [17,18]:

*P*_0_/*P* × (1 − ϕ) = (1 + αϕ/3)^2^ (1)

where *P*_0_ is the permeability parameter of the pure biopolymer coating, *P* is the permeability parameter of the bionanocomposite coatings, α is the aspect ratio of the platelets (the width divided by the thickness), and ϕ is the volume fraction of the platelets dispersed in the biopolymer matrix. Experimental *OTR* data at 30% RH is compatible with Cussler’s prediction for α = 4000 (Figure 2a), up to a concentration of the filler that is equal to 0.2 wt %, namely ϕ = 0.00077. This clearly suggests that Cussler’s model approached the best fit of experimental data at α = 4000 for mica, which is in line with our experimental values obtained by AFM.

Above the 0.2 wt % (ϕ = 0.00077) threshold, a clear deviation was observed between experimental and predicted *OTR* values, which can be reasonably due to the aggregation and stacking of mica sheets at high loading, according to the well-known “self-similar clay aggregation mechanism” [19]. This aspect is further emphasized as the relative humidity increased. At 90% RH, the applied model underestimated the aspect ratio of the filler to be lower than 100, which is in clear contrast with the TEM and AFM observations. This unrealistic prediction can be explained once again in terms of aggregation of the filler, which is exaggerated due to the aforementioned diluting effect of water molecules, insomuch as the tactoid configuration of mica can be thought to be restored.

The model simulation at lower RHs, together with the AFM and TEM results, strongly supports the higher aspect ratio of mica, compared to the widely used inorganic clays (e.g., montmorillonite), which have been reported to have aspect ratios between 10 and 100 for a similar pullulan-based system [9,20]. This aspect turns out to be of great importance during the design of high performance barrier materials (e.g., films and coatings), since using mica would be, in principle, more effective than using clays due to the wider surface (for the same volume fraction) opposed to the permeation.

### 2.3. Mechanical Properties

#### 2.3.1. Friction Behavior

Control of friction influences a broad range of material applications, such as the packaging industry. Here, low coefficients of friction are desirable in order to avoid blocking the plastic webs that run on packaging lines, which would eventually affect the throughput of the process. For this reason, the presence of nanocoatings that are deposited on the plastic surface may have a relevant impact on the friction behavior of the plastic materials. A few studies have assessed the inherent friction behavior of mica layers [21,22]. However, no studies that investigated the improvement of the frictional performance of mica-based polymeric materials have been found.

Both static (*µ*_s_) and dynamic (*µ*_k_) friction coefficients of PET, pullulan, and pullulan-mica coatings are reported in Table 2. The addition of mica significantly decreased both *μ*_s_ and *μ*_k_ at a concentration of 0.2 wt %. In particular, a decrease of approximately 12% and 23% for the two coefficients, respectively, was obtained compared to the neat PET. Comparatively, a reduction of ≈26% for both coefficients was achieved compared to the pullulan-coated PET. The explanation for the observed improvement lies in the surface morphology of the nanocomposite coatings. More specifically, the addition of mica roughened the surface of the coatings in comparison to the unloaded pullulan coating, which exhibited a surface roughness (expressed as root-mean-square roughness, or RMS) between 1.2 nm and 3.0 nm [9,23]. In this work, RMS values of 4.0 nm and 7.0 nm were obtained by AFM for the pullulan coatings loaded with mica at concentrations of 0.2 wt % and 1.5 wt %, respectively. Noticeably, increasing the concentration of mica loaded in the main pullulan matrix to be above 0.2 wt % did not result in any appreciable improvement in the friction performance; that is, the loading of 0.2 wt % (ϕ = 0.00077) was the minimum and sufficient amount to achieve the reduction in both friction coefficients. Reversely, the fact that concentrations of the filler below 0.2 wt % had no effect on the friction performance is plausibly explained by considering that the tribological behavior of the coatings surface is influenced by the surface morphology and topography at a macro-scale level (i.e., when filler aggregates form on the coating surface). As already reported, differences at the nano-scale level do not seem to have any significant effect [24,25].

#### 2.3.2. Tensile Properties

Mechanical properties of uncoated PET, pullulan-coated PET, and bionanocomposite films with different amounts of mica are summarized in Table 2. In general, the use of clay nanoplatelets as a reinforcing agent in polymeric matrices leads to an increase in stiffness and a concomitant decrease in elongation [26]. However, few studies can be found on the effect of nanocomposite coatings on the tensile properties of the plastic substrate beneath. In this work, the nanocomposite coating deposited on the PET substrate had a significant effect from a mica concentration of 0.2 wt % and greater. The elastic modulus (Emod) increased linearly, with a mica concentration up to a 7 wt % increase at a mica loading of 1.5 wt %, compared to the neat PET and pullulan-coated PET.

Despite the high stiffness of mica platelets (≈50 GPa) [27], the final reinforcing effect of mica was indeed moderate. The reason is that mica was added only to the coating biopolymer matrix (i.e., pullulan), while the ultimate elastic modulus value accounts for the final material, which includes the thin coating (less than 1 μm) and the thicker plastic substrate (≈12 μm).

That is, the greatest contribution on the final performance comes from the substrate, rather than the coating. For the same reason, the elongation at break (%) was not significantly influenced by the addition of synthetic mica to the pullulan coating. As for the tensile strength (TS), a significant improvement was recorded only at a concentration of 0.2 wt %, whereas at higher mica loadings TS decreased again, thus approaching the original values of bare PET and pullulan-coated PET. This behavior is most likely related to stress concentration points at the sharp tactoid edges, resulting in flaws (i.e., mechanical failures) at the polymer/filler interface, as already observed in poly(methyl methacrylate) (PMMA)/clay [27], poly(ethylene terephthalate)/mica [4], and epoxy/clay [7] nanocomposites.

### 2.4. Optical Properties

The optical properties of materials are of great importance for a large number of applications, such as construction (e.g., reflective glasses), agriculture (e.g., greenhouse windows), energy (e.g., solar cells), display/screen, and food packaging, just to provide a few examples. In many applications, transparent materials are sought in order to have a clear vision of the objects. From a practical point of view, the “see-through” property, which is obtained by reducing the contrast between objects viewed through the material, is actually one of the most important requirements as it can influence the final choice made by consumers [28].

For all of the coating formulations, the haze value of the coated PET is within 3.0 wt %, with the lowest value recorded for the pullulan-coated PET (Table 3). The 3.0 wt % threshold is deemed necessary to warrant a suitable display of the item behind the plastic film [9]. The only exception is represented by the formulation that includes the highest amount of mica (1.5 wt %), for which a final haze of 3.23% was measured. The increase in haze observed for films and coatings can be explained in terms of surface roughness [29] and the presence of scattering centers that come from the reaggregation of the nanoparticles [9]. As discussed previously (see Section 2.3.1), the addition of mica led to an increase in the roughness of the coating surface, in particular for the highest mica concentration (RMS = 7.0 nm). This, along with the reaggregation phenomenon postulated for the high mica concentrations (see Section 2.2), would justify the slight increase in haze arising from the addition of the filler. This observation is consistent with previous works on nanocomposite polymers, including platy [9,10,21] and rod-shape [11] nanoparticles.

Transmittance values for uncoated PET and coated PET are reported in Table 3. All of the coating formulations had transmittance values that were higher than the bare PET, meaning that the coating deposition improved the overall performance of the plastic substrate. In particular, the best performance was again recorded for the pullulan-coated PET, thus suggesting the “clarity-enhancer” attribute of this biopolymer, presumably due to anti-reflective properties of this polysaccharide in the form of thin layers. To confirm this, we have decided to carry out some ellipsometry experiments.

In an initial step, the plastic substrate alone has been characterized to gather a proper model enabling the simulation of the PET optical properties.

The ellipsometry results were fitted by assuming a semi-infinite bulk of a transparent material with a refractive index described by the Cauchy model:

*n*(λ) = *n*_A_ + *n*_B_/λ^2^ (2)

where *n*_A_ and *n*_B_ are free parameters for the fitting. Experimental evidence was found of interference fringes attributed to a possible thin layer on top of the PET substrate. However, this layer was neglected in the model since its effects were not critical and a reliable fitting of the ellipsometry data were obtained with the simpler model of a semi-infinite bulk material. The fitting procedure provided *n*_A_ = 1.8564 and *n*_B_ = 0.0019896, corresponding to a mean refractive index of the PET substrate in the visible range (320–700 nm) equal to 1.864 ± 0.002. The correlation factor between the *n*_A_ and *n*_B_ obtained by the fitting was relatively large (94.3%). Nevertheless, the model can be considered reliable because: (i) the correlation between *n*_A_ and *n*_B_ did not strongly affect the mean refractive index in the visible range; and (ii) the obtained refractive index is in reasonable agreement with data reported in the literature [30], notwithstanding the adopted approximation of a simplified model.

**Figure 3:**
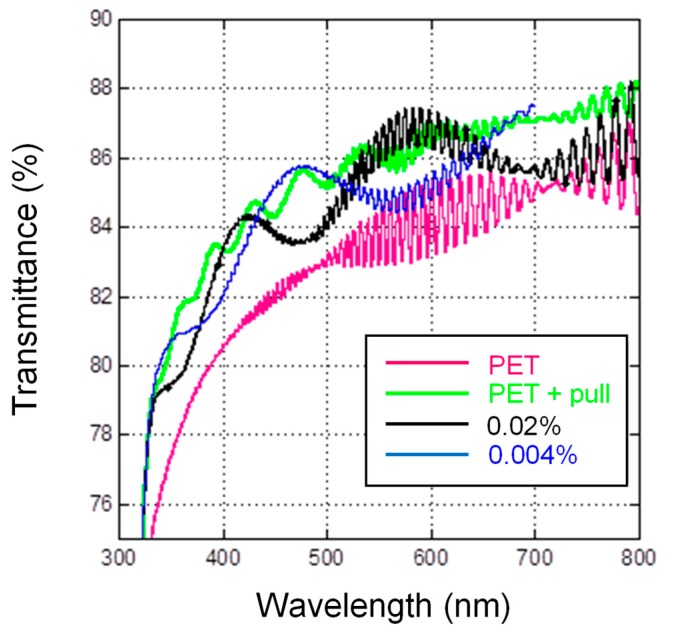
Transmittance spectra of bare PET, pullulan-coated PET, and PET coated with the nanocomposite coatings at different concentrations of mica (0.02 wt % and 0.04 wt %).

The model obtained for the PET semi-infinite substrate was then used to investigate the optical properties of the pullulan coating laid on the PET surface. The stack for the simulation was built by adding a transparent layer on top of the PET semi-infinite substrate. The optical properties of the PET were assumed to be known, as deduced from the previous ellipsometry measurements. The optical properties of the added layer were described by the Cauchy model, with new *n*_A_ and *n*_B_ parameters. The fitting of the ellipsometry data was performed with four free parameters, namely *n*_A_ and *n*_B_, the thickness *t* of the added layer, and its thickness non-uniformity *t*_n-u_. The results of the fitting are reported in the first line of Table 4, together with the corresponding mean-squared-error (*MSE)* value of the fitting and the refractive index of the top layer. The mean refractive index is in reasonable agreement with data reported in the literature for pullulan [31]. The correlation factors between the free parameters of the fitting are reported in Table S1. The relatively large correlation (67.9%) between the parameters *n*_A_ and *n*_B_ of the Cauchy model does not invalidate the result, for the same reasons as discussed for the PET substrate. The other correlation factors are relatively low. The same procedure and model were also adopted for the pullulan coating loaded with the lowest amount of mica (0.02 wt %). It is noticed that the lowest and highest refractive index is recorded for the uncoated PET and the pullulan-coated PET, respectively. Similarly, the non-uniform thickness of the top layer increased with the addition of mica. Transmittance measurements in the 320–800 nm spectral range were also performed at normal incidence on the PET, PET-pullulan, and PET-pullulan samples loaded with 0.02 wt % and 0.04 wt % of mica (Figure 3). The PET substrate showed a relatively low transmittance, while a marked increase was detected for the PET film coated with pullulan. This further demonstrates the anti-reflection behavior of the pullulan layer. Indeed, the refractive index of pullulan is intermediate between air and PET. With the addition of mica, the refractive index of the top layer increased again, approaching the PET refractive index value. Therefore, the addition of the nanofiller reduced the original anti-reflection behavior of the pullulan coating. This is confirmed by the fact that the transmittance spectra of the PET films coated with the nanocomposite formulations are half-way placed, with the spectrum obtained for the highest concentration of mica (0.02 wt %) closer to the transmittance spectrum of the bare PET.

The mean value of the transmittance measured in the visible region is reported in Table 3. Transmittance was also simulated by taking into consideration the model and the parameters obtained by ellipsometry for each sample (T_simul_ of Table 3).

A good agreement was found between the measured and simulated values. The lowest transmittance was found for the PET substrate, while the largest one was again recorded for the pullulan-coated PET sample. The presence of mica in the top layer determined a decrease in the transmittance. Even if the reflectance was not experimentally measured, the spectra were calculated for near-normal incidence (20°, polarization *s*), based on the ellipsometry models (Table 3, last column). As expected, the minimum reflectance was calculated for the pullulan-coated PET sample, due to the anti-reflection behavior of the pullulan layer. Comparatively, an increase was observed when increasing the concentration of mica, which is consistent with previous measurements. These results confirm the anti-reflective properties of pullulan. The same behavior was described in previous works on pullulan-coated bi-oriented polypropylene (BOPP) [24] and low-density polyethylene (LDPE) [28]. The reason for this unique property of pullulan (compared to other biopolymers) lies in its fully amorphous organization, which in turn must be ascribed to its inherent structural flexibility centered on the α-(1→6)-linkage between maltotriose units [13].

## 3. Materials and Methods

### 3.1. Raw Materials and Reagents

Pullulan (PI-20 grade, M_w_ ≈ 200 kDa) was purchased from Hayashibara Biochemical Laboratories Inc. (Okayama, Japan), which currently belongs to the Nagase Group. The structural characteristics of pullulan (determined by high-performance size-exclusion chromatography equipped with multi-laser scattering and refractive index detectors—HPSEC-MALLS-RI) are weight average molar mass (M_w_) = 2.094 × 10^5^ ± 0.002; polydispersity index (M_w_/M_n_) = 1.321 ± 0.02; and radius of gyration (*R*_g_) = 24.7 ± 0.002 nm [32]. Synthetic swelling type mica NTS-5 (Na-tetrasilic mica, NaMg_2.5_Si_4_O_10_F_2_) was purchased as a water dispersion (6 wt %) from Topy Industries Ltd. (Toyohashi, Japan). As a plastic substrate, AryaPET–A410 (JBF RAK LLC, Ras Al Khaimah, United Arab Emirates), kindly provided by Metalvuoto Spa (Roncello, Italy), was used. It is a one-side corona-treated polyester film 12.0 ± 0.5 μm thick, suitable for metallizing, printing, and lamination, with good wettability and excellent machinability. Milli-Q water (18.3 MΩ cm) was used as the only solvent throughout the experiments.

### 3.2. Preparation of the Bionanocomposite Coatings

A fixed amount of pullulan (10 wt %, wet basis) was dissolved in distilled water at 25 °C for 1 h under gentle stirring (500 rpm). Afterward, 50 mL of the mica dispersion (0.2 wt %, wet basis) was ultrasonicated by means of an UP400S (maximum power = 400 W; frequency = 24 kHz) ultrasonic device (Hielscher, Teltow, Germany), equipped with a cylindrical titanium sonotrode (mod. H14, tip Ø 14 mm, amplitude_max_ = 125 μm; surface intensity = 105 W cm^−2^) under the following conditions: 0.5 cycle and 50% amplitude for 2 min. In parallel, the resulting mica dispersions were diluted in distilled water (18.3 MΩ cm) under vigorous stirring (500 rpm) for 15 min. The pullulan solution and the inorganic dispersion were then mixed together under gentle stirring (300 rpm) for an additional 60 min. More specifically, the quantity of mica in the pullulan-water solutions was 0.002, 0.004, 0.006, 0.008, 0.01 and 0.02 wt % (wet basis). After drying, the concentrations of mica corresponded to 0.02, 0.04, 0.06, 0.08, 0.1 and 0.2 wt % on dry basis. PET films were treated with a high frequency corona treatment (Arcotec, Ülm, Germany). An aliquot of each bionanocomposite water dispersion was then placed on the corona-treated side of rectangular (24 × 18 cm^2^) PET samples. The deposition of the coating was carried out by using an automatic film applicator (ref 1137, Sheen Instruments, Kingston, UK) at a constant speed of 2.5 mm s^−1^, according to ASTM D823-07—Practice C. The deposition was performed by using a horizontal steel rod with an engraved pattern, which yielded final coatings of comparable nominal thickness of 1 µm after water evaporation. Water evaporation was performed using a constant and perpendicular flux of mild air (25.0 ± 0.3 °C for 2 min) at a distance of 40 cm from the applicator. The coated films were then stored under controlled conditions (23.0 ± 0.5 °C in a desiccator) for 48 h before measurements.

### 3.3. Analyses

The thickness of the pullulan/mica nanocomposite coating was obtained by a gravimetric method. A 10 × 10 cm^2^ sample (coated PET) was cut and weighed (M_1_, grams). The coating was then mechanically removed by immersion in hot water (80 °C), and the resulting bare PET film was weighed (M_2_, grams). The apparent thickness (μm) of the coating was obtained according to the following equation [33]:

*l* = [(M_1_ − M_2_)/ρ] × 100 (3)

where ρ (g cm^−3^) is the density of the aqueous dispersion. Three replicates were analyzed for each biopolymer composition.

Mica nanosheets were characterized by both TEM and AFM. The TEM images were acquired by using a LEO 912 AB energy-filtering transmission electron microscope (EFTEM) (Carl Zeiss, Oberkochen, Germany) operating at 80 kV. Digital images were recorded with a ProScan 1K Slow-Scan CCD camera (Proscan, Scheuring, Germany). Samples for TEM analyses were prepared by drop-casting a few millilitres of dispersion onto Formvar-coated Cu grids (400-mesh). The samples rested for 24 h at room temperature to allow water to evaporate. The AFM experiments were carried out to quantify the size features of mica nanosheets (e.g., width and thickness). The analyses were performed with a Nanoscope V Multimode (Bruker, Karlsruhe, Germany) in intermittent-contact mode after dropping 10 μL of diluted mica water dispersion (0.2 mg mL^−1^ and 0.02 mg mL^−1^) onto a mica substrate. The images were collected with a resolution of 512 × 512 pixels with silicon tips (force constant 40 N m^−1^, resonance frequency 300 kHz). Dimensional calculations on the acquired images were conducted with nanoscope software (version 7.30, Bruker, Karlsruhe, Germany). The mean values reported for mica sheet dimensions were calculated over several images.

The oxygen barrier properties of the films were assessed on a 50 cm^2^ surface sample using a Multiperm permeability analyzer (Extrasolution Srl, Capannori, Italy) equipped with an electrochemical sensor. The *OTR* data were determined according to the standard method of ASTM F2622-08, with a carrier flow (N_2_) of 10 ml min^−1^ at 23 °C at 0%, 30%, 60%, and 90% relative humidity (RH) and at 1 atm pressure difference on the two sides of the specimen. During the analyses, the coated side of each sample faced the upper semi-chamber into which the humid test gas (oxygen) was flushed. Each *OTR* value was from three replicates.

Static (*µ*_s_) and kinetic (*µ*_k_) friction coefficients were measured using a dynamometer (model Z005, Zwick Roell, Ulm, Germany), in accordance with the standard method ASTM D 1894-87. The software TestXpert V10.11 (Zwick Roell, Ulm, Germany) was used for data analysis. The friction opposing the onset on relative motion (impending motion) is represented by *µ*_s_, whereas *µ*_k_ can be considered as the friction opposing the continuance of the relative motion once that motion has started. In the case of solid-on-solid friction (with or without lubricants), these two types of friction coefficients are conventionally defined as follows:

*µ*_s_ = *F*_s_·*P* (4a)

*µ*_k_ = *F*_k_·*P* (4b)

where *F*_s_ is the force just sufficient to prevent the relative motion between two bodies, *F*_k_ is the force needed to maintain the relative motion between the two bodies and *P* is the force normal to the interface between the sliding bodies. In this study, the motion of each type of film (coated and uncoated) on a metallic rigid surface (a polished stainless steel 150 × 450 × 3 mm^3^) was considered. This surface, in addition to acting as a supporting base to guarantee a firm position between the moving crosshead and the force-measuring device, served the purpose of simulating the friction between the plastic web and the metallic parts of the equipment used during the manufacturing processes and operations.

Tensile properties of films were measured according to the ASTM D882-02 by means of a dynamometer (mod. Z005, Zwick Roell, Ulm, Germany) fitted with a 5 kN load cell and connected with two clamps placed at 125 mm apart. Elastic (Young’s) modulus (Emod), tensile strength (TS), and elongation (ε) were gathered from the stress-strain curves. For each parameter, the final results are the mean of at least five replicates.

Transparency and haze were determined by using a UV-Vis, high-performance spectrophotometer (Lambda 650, PerkinElmer, Waltham, MA, USA). Transparency was assessed in terms of specular transmittance (i.e., the transmittance value obtained when the transmitted radiant flux includes only the light transmitted in the same direction as that of the incident flux at a 550 nm wavelength) in accordance with the ASTM D1746-88.

Haze was measured within the wavelength range of 780–380 nm, in accordance with ASTM D1003-00, by using a 150-mm integrating sphere coupled with the main spectrophotometer, in order to trap the diffuse transmitted light. Haze is defined as the percentage of transmitted light deviating by more than an angle of 2.5° from the direction of the incident beam. Three replicates were made for each uncoated and coated film sample.

Variable-angle spectroscopic ellipsometry (VASE) measurements were performed in the spectral range from 320 nm to 800 nm (with steps of 2 nm), at different angles of incidence (from 40° to 70°), using the ellipsometer J.A. Woollam Co. Inc. (Lincoln, NE, USA). The ratio between the elements of the Jones matrix (r_pp_/r_ss_) = tan (Ψ) e^iΔ^ were acquired through Ψ and Δ, which depend on the wavelength λ of the incident polarized light. The measured Ψ(λ) and Δ(λ) data were analyzed with the software WVASE 32 by describing the sample using the following model. The fitting of the experimental data was performed by minimizing the mean-squared-error (*MSE*) defined as:

(5) $$MSE=\sqrt{\frac{\sum_{i=1}^{N}\left[\left(\frac{\psi_{i}^{\mathrm{mod}}-\psi_{i}^{\exp}}{\sigma_{\psi ,i}^{\exp}}\right)+\left(\frac{\Delta_{i}^{\mathrm{mod}}-\Delta_{i}^{\exp}}{\sigma_{\Delta ,i}^{\exp}}\right)\right]}{2N-M}}$$

where *N* is the number of measured Ψ and Δ pairs and *M* is the total number of model fit parameters.

The statistical significance of differences was determined by one-way analysis of variance (ANOVA), using JMP 5.0.1 software (SAS, Cary, NC, USA). Where appropriate, the mean values were compared by using a least significant difference (LSD) test, with a significance level *p* < 0.05.

## 4. Conclusions

The fabrication of water-based nanocomposite coatings incorporating mica NBBs into pullulan has been proposed in this study as a feasible and environmentally friendly process. Besides the exceptional oxygen barrier performance (especially at low and middle RHs), the PET films with the nanocomposite coatings exhibited outstanding optical properties, even at high loadings of mica. Interestingly, we have demonstrated that the addition of the nanocomposite coating moderately improved the elastic modulus and friction properties of the final material. The findings that arise from this work suggest the use of mica as a valid alternative to more common (e.g., montmorillonite) or more appealing (e.g., graphene and its derivatives) nanosheets for the design of nanocomposite coatings of potential utility in different fields.

## Figures and Tables

**Table 1:** Oxygen transmission rate (*OTR*) of uncoated poly(ethylene terephthalate) (PET) and coated PET and bionanocomposite coatings at 0%, 30%, 60%, and 90% relative humidity (RH) for the different filler volume fraction (ϕ).

Mica Content		*l* (µm)	*OTR* (mL m^−2^ 24 h^−1^)			
wt %	ϕ ^†^		0% RH	30% RH	60% RH	90% RH
uncoated PET	-	12.00 ± 0.03 ^b^	129.23 ± 2.6 ^a^	120.67 ± 0.9 ^a^	115.10 ± 2.76 ^a^	107.47 ± 0.74 ^1^
PET/pullulan	-	12.75 ± 0.07 ^a^	5.99 ± 0.02 ^b^	26.74 ± 0.3 ^b^	45.80 ± 2.65 ^bc^	100.73 ± 3.23 ^cd^
0.02	0.00008	12.76 ± 0.06 ^a^	1.27 ± 0.24 ^c^	18.79 ± 2.23 ^c^	51.21 ± 4.98 ^b^	100.63 ± 1.45 ^cd^
0.04	0.00015	12.75 ± 0.07 ^a^	1.12 ± 0.29 ^c^	14.09 ± 2.74 ^d^	50.20 ± 4.28 ^b^	100.79 ± 4.27 ^cd^
0.06	0.00023	12.78 ± 0.01 ^a^	N.D.	13.35 ± 0.51 ^d^	50.94 ± 0.1 ^b^	99.56 ± 0.63 ^cd^
0.08	0.00031	12.80 ± 0.05 ^a^	N.D.	11.18 ± 2.24 ^de^	49.10 ± 0.31 ^b^	97.47 ± 0.42 ^d^
0.1	0.00038	12.76 ± 0.05 ^a^	N.D.	10.29 ± 0.3 ^e^	46.76 ± 1.1 ^bc^	96.62 ± 0.58 ^d^
0.2	0.00077	12.77 ± 0.05 ^a^	N.D.	8.39 ± 0.44 ^ef^	41.05 ± 0.65 ^cd^	102.40 ± 1.17 ^bc^
0.5	0.00256	12.75 ± 0.01 ^a^	N.D.	6.05 ± 0.48 ^f^	36.01 ± 3.97 ^d^	101.11 ± 0.71 ^cd^
1	0.00510	12.80 ± 0.06 ^a^	N.D.	6.89 ± 0.6 ^f^	35.56 ± 5.6 ^d^	105.88 ± 3.28 ^ab^
1.5	0.01141	12.77 ± 0.06 ^a^	N.D.	8.50 ± 0.6 ^ef^	24.95 ± 1.16 ^e^	103.22 ± 2.04 ^abc^

**^†^** Calculated for a given mica density (ρ) = 2.6 g cm^−3^ and pullulan density (ρ) = 1 g cm^−3^. ^abcdef^ Different superscripts within a group (i.e., within each parameter) denote a statistically significant difference (*p* < 0.05). Error around the mean value represents the standard deviation. N.D.: below the instrument detection limit (<0.01 mL m^−2^ 24^−1^).

**Table 2:** Coefficient of friction (COF, static and dynamic), elastic modulus (Emod), elongation at break (ε), and tensile strength (TS) of uncoated PET, pullulan-coated PET, and bionanocomposite coatings for different mica concentrations (wt %).

Sample	COF (Coating/Metal)		Emod (GPa)	ε (%)	TS (MPa)
	*µ*_s_	*µ*_k_			
uncoated PET	0.35 ± 0.01 ^a^	0.26 ± 0.02 ^a^	3.65 ± 0.20 ^a^	15.80 ± 3.23 ^a^	104.99 ± 9.65 ^ab^
PET/Pullulan	0.42 ± 0.02 ^b^	0.27 ± 0.01 ^a^	3.65 ± 0.15 ^a^	15.52 ± 3.50 ^a^	105.33 ± 8.98 ^ab^
Mica 0.02%	0.35 ± 0.01 ^a^	0.23 ± 0.01 ^b^	3.63 ± 0.15 ^a^	16.37 ± 1.67 ^a^	109.43 ± 2.33 ^ab^
Mica 0.2%	0.31 ± 0.02 ^c^	0.20 ± 0.02 ^c^	3.71 ± 0.12 ^ab^	18.82 ± 5.16 ^a^	113.45 ± 7.04 ^a^
Mica 0.5%	0.31 ± 0.02 ^c^	0.20 ± 0.01 ^c^	3.79 ± 0.17 ^ab^	18.61 ± 2.99 ^a^	105.23 ± 7.88 ^ab^
Mica 1.0%	0.31 ± 0.02 ^c^	0.21 ± 0.02 ^bc^	3.81 ± 0.06 ^ab^	18.97 ± 2.68 ^a^	105.51 ± 10.23 ^ab^
Mica 1.5%	0.31 ± 0.01 ^c^	0.20 ± 0.01 ^c^	3.90 ± 0.20 ^b^	16.79 ± 3.15 ^a^	102.21 ± 9.81 ^b^

^abc^ Different superscripts within a group (i.e., within each parameter) denote a statistically significant difference (*p* < 0.05). Error around the mean value represents the standard deviation.

**Table 3:** Haze (H), transmittance (T), and reflectance (R) of uncoated PET, pullulan-coated PET, and bionanocomposite coatings for different mica concentrations (wt %).

Sample	H (%)	T (%) 550 nm	T (%) 400–700 nm ^†^	T_simul_^†^	R (%) 400–700 nm ^†^
uncoated PET	2.72 ± 0.08 ^bc^	82.88 ± 0.77 ^a^	83.4 ± 1.3	83.3 ± 0.1	10.4 ± 0.1
PET-pullulan	2.63 ± 0.22 ^bc^	86.30 ± 0.94 ^b^	86.9 ± 1.0	86.4 ± 1.2	6.4 ± 1.6
Mica 0.04%	2.81 ± 0.21 ^b^	85.04 ± 0.41 ^b^	N.A.	N.A.	N.A.
Mica 0.02%	2.69 ± 0.11 ^bc^	85.72 ± 1.02 ^b^	85.3 ± 1.2	84.9 ± 0.3	8.4 ± 0.4
Mica 1.5%	3.23 ± 0.17 ^a^	83.28 ± 0.32 ^a^	N.D.	N.D.	N.D.

^†^ By ellipsometry. ^abc^ Different superscripts within a group (i.e., within each parameter) denote a statistically significant difference (*p* < 0.05). Error around the mean value represents the standard deviation. N.D.: not determined.

**Table 4:** Parameters *n*_A_, *n*_B_, *t*, and *t*_n-u_ of the top layer obtained by fitting the ellipsometry Ψ(λ) and Δ(λ) experimental data, *MSE* of the fitting, and deduced refractive index of the top layer both at 589 nm (*n*_589_) and averaged in the visible range from 400 nm to 700 nm (*n*_mean_ ± std dev).

Samples	*n*_A_	*n*_B_ (μm^2^)	*t* (nm)	*t*_n-u_ (%)	*MSE*	*n*_589_	*n*_mean_ ± std dev
uncoated PET	1.8564	0.0019896	-	-	-	-	1.864 ± 0.002
PET-pullulan	1.5593	0.0020923	470.50	20.6	4.71	1.559	1.569 ± 0.002
Mica 0.04 wt %	1.6552	0.021063	465.53	41.6	1.58	1.716	1.731 ± 0.026

## Supplementary Materials

The following are available online at http://www.mdpi.com/2079-4991/7/9/281/s1, Table S1: Correlation factors between the free parameters of the fitting of the ellipsometry data for the different samples.
