# Supplementary material for: Transparent Pullulan/Mica Nanocomposite Coatings with Outstanding Oxygen Barrier Properties
Source: Nanomaterials (Basel). 2017 Sep 19;7(9):281. doi: 10.3390/nano7090281 (PMC5618392; doi:10.3390/nano7090281)
Supplement: Supplementary file 1 [file nanomaterials-07-00281-s001.pdf]

Supporting Information

Table S1. Correlation factors between the free parameters of the fitting of the ellipsometry data for the different samples.

**Sample**	**Correlation factors**	n_A	n_B (μm²)	t (nm)	t_{n-u} (%)
PET	n_A	-	94.3%	-	-
	n_B (μm²)		-	-	-
	t (nm)			-	-
	t_{n-u} (%)				-
Pullulan	n_A	-	67.9%	33.5%	46.9%
	n_B (μm²)		-	31.7%	2.2%
	t (nm)			-	44.4%
	t_{n-u} (%)				-
Pullulan/mica 0.2 wt. %	n_A	-	70.4%	4.3%	27.6%
	n_B (μm²)		-	2.2%	64.8%
	t (nm)			-	61.4%
	t_{n-u} (%)				-
